# Association between migraine severity and sleep quality: a nationwide cross-sectional study

**DOI:** 10.3389/fneur.2025.1529213

**Published:** 2025-01-31

**Authors:** Nura A. Almansour, Seham S. Alsalamah, Razan S. Alsubaie, Nada N. Alshathri, Yasmeen A. Alhedyan, Faisal Y. Althekair’s

**Affiliations:** ^1^College of Medicine, King Saud bin Abdulaziz University for Health Sciences, Riyadh, Saudi Arabia; ^2^King Abdullah International Medical Research Center (KAIMRC), Riyadh, Saudi Arabia; ^3^Department of Neurology, Ministry of National Guard - Health Affairs, Riyadh, Saudi Arabia

**Keywords:** sleep quality, migraine, Pittsburgh Sleep Quality Index, migraine disability assessment scale, association, migraine severity, sleep disorders, migraine prevalence

## Abstract

**Background:**

Migraine is a primary headache disorder that affects more than 1 billion individuals globally and imposes a significant disability burden on society. Although migraine patients commonly experience poor sleep quality, the relationship between migraine and sleep is not yet fully understood. This study therefore aimed to determine the association between sleep quality and migraine severity.

**Methods:**

A comparative cross-sectional study was conducted with 1,399 participants across all regions of Saudi Arabia from August to October 2023 using standardized questionnaires. Participants were categorized into patients with migraine and non-migraine patients, according to the International Headache Society (IHS) criteria. This study utilized The Migraine Disability Assessment Scale (MIDAS) and Pittsburgh Sleep Quality Index (PSQI) to evaluate migraine severity and sleep quality, respectively.

**Results:**

The prevalence of migraine was 25%, while poor sleep quality was evident in 42.4% of the patients. No significant difference in PSQI scores was observed between patients with migraine and non-migraine patients (*p* = 0.821). Migraine patients with poor sleep quality showed significantly higher MIDAS scores than those with good sleep quality (10.37 vs. 6.58; *p* = 0.002), while patients with migraine with higher levels of disability had higher PSQI scores than those with lower levels of disability, although the difference was not statistically significance (7.61 vs. 6.81, *p* = 0.053). A significant positive correlation was found between the PSQI and MIDAS scores (*r* = 0.179, *p* < 0.001). MIDAS was also significantly positively correlated with the following PSQI components: subjective sleep quality (*p* = 0.047), sleep latency (*p* < 0.001), sleep disturbance (*p* < 0.001), and daytime dysfunction (*p* < 0.001).

**Conclusion:**

These findings suggest a notable correlation between poor sleep quality and increased migraine severity, emphasizing the importance of addressing sleep disturbance as a potential strategy to mitigate migraine severity and improve patient outcomes.

## Background

Migraine is a recurrent, burdensome neurovascular disorder characterized by bouts of disabling, throbbing, and often unilateral headaches, accompanied by various concomitant symptoms such as nausea, vomiting, and light and sound sensitivity (1). Of all headache disorders, migraine is one of the most prevalent and most disabling (2). Globally, migraine ranked as the 19th most common cause of disability according to the Global Burden of Disease Report (GBD) in 2000 (3). However, on the GBD 2015, migraine was ranked as the third most disabling disorder in individuals below 50 years of age (4). Along the same lines, the GBD Report 2016 shows that tension-type headaches and migraine are ranked third and sixth in terms of prevalence, respectively (2). Although the underlying pathophysiology has not yet been fully elucidated, migraine are known to be triggered by numerous environmental factors, including lifestyle, hormones, and sleep disturbances (5). Sleep is a physiological aspect of human life that is essential for preserving health and welfare. It is a natural and reversible condition primarily regulated by neurobiological processes (6, 7). Sleep quality is a multifaceted construct that considers several factors, including waking following sleep start, frequency and number of night awakenings, sleep latency, and subjective assessments of rested and awakened (8). A “good night’s sleep” is generally classified as a night with adequate rest (often 78 h) to allow for the homeostatic restorative process, and it is distinguished by an overall high quality of the entire sleep period (9). One-third of the general population experienced sleep problems. Sleep quality is generally considered as a significant indicator of quality of life, given the high prevalence of sleep disorders and the close connection between sleep and quality of life (10).

Poor sleep quality is more common in patients with migraine than in the general population and affects migraine patients in many ways (11). In one study investigating 103 university students with migraine, 85 (82.5%) had poor sleep quality, whereas 18 (17.5%) had normal sleep quality (12). In another study, one-third of 50 patients with migraine and tension-type headaches reported sleep problems as the cause of their headaches. Of these, 68.7% were awakened by headaches, 30% of whom reported that their headaches were caused by sleep deprivation, excessive sleep, or both (13). Migraine chronicity is significantly affected by poor sleep quality (9, 14). It is also possible that migraine episodes were preceded by poor sleep (15). Headache severity and frequency are commonly affected both directly and indirectly by sleep quality in migraine (16). Similarly, because migraine has frequently been observed as a triggering factor, they can also be a triggering factor for other types of illnesses. Migraine patients have an increased risk of ischemic stroke, sleep disruption, and depression (17–19). Further, sleep quality and how migraine exerts its effects have been emphasized owing to their relevance. A high incidence of 61.61% of patients with migraine complain about their sleep quality (20). Furthermore, the relationship between migraine and sleep has been validated by the prevalent complaints regarding difficulty in initiating and maintaining a healthy sleep rhythm (21). Moreover, sleep interruption is a common problem among patients with migraine, and multiple studies have shown a higher prevalence of poor sleep quality compared with normal individuals (22–25). Although Fox and Davis concluded that headache attacks are not associated with disturbed sleep (26), while indistinguishable sleep parameters were reported in migraine patients and controls, migraine subjects exhibited a longer sleep onset latency (27). Boosting sleep quality has also shown to be more achievable if the recurrence of headache attacks diminishes (28). These findings indicate that a complex relationship exists between migraine and sleep quality.

Migraine patients have further been found to have reduced subjective sleep quality and altered sleep architecture compared to non-migraine patients (29). However, data on the association between migraine and sleep quality are lacking. The present study is one of the first to thoroughly examine the relationship between migraine and sleep quality from a population-based perspective to learn more about the specific relationship between migraine and sleep quality.

## Methods

### Subjects

This study investigated the association between migraine severity and sleep quality in all regions of Saudi Arabia from August to October 2023. This study is a comparative cross-sectional study enrolling 1,399 participants from all regions of Saudi Arabia. The required sample size was calculated using the Epitools website^1^ using g-power, with an effect size of 0.1 and one degree of freedom. The inclusion criterion was anyone above the age of 18 years of either sex. The exclusion criteria included primary headaches other than migraine, evaluation using the IHS criteria, and surgery within the last 6 months. The participants were further divided into migraine patients (cases) and non-migraine patients (controls), based on the application of the IHS criteria.

### Sociodemographic data form

Data were obtained using an online, structured, self-administered questionnaire in Arabic created using Google Forms. The first part of the questionnaire explored the sociodemographic characteristics of the study participants. Age, sex, height, weight, and BMI of the participants were all obtained. Other participant characteristics, such as smoking status, presence of comorbidities, and family history of headaches, were also recorded. Finally, a screening question regarding the history of headaches in one’s life was posed to all participants. If the participant answered yes, they were moved to the IHS criteria page and the Migraine Disability Assessment Scale (MIDAS). If not, they would move on to the Pittsburgh Sleep Quality Index (PSQI) page.

### Assessment of migraine

The International Headache Society (IHS) criteria were included in the questionnaire to diagnose and differentiate patients with migraine from non-migraine patients (control group). The IHS classification is a 4-digit hierarchical method used to diagnose headaches with variable specificities, with the first digit being specific for diagnostic categories, the second digit for subtypes, and the third and fourth digits for more specifications. The criteria were as follows: untreated or unsuccessfully treated headache attacks that last 4–72 h occurring more than 15 times per month not attributed to any other disease; a headache that had at least two features (unilateral, pulsatile, moderate, and severe pain, aggravated by physical activity); and at least one of the following associated symptoms (nausea and/or vomiting, photophobia, or phonophobia) must occur alongside the headache (30).

### Migraine disability assessment scale

An Arabic-validated form of the MIDAS questionnaire was used to measure disability levels linked to migraine (31). Comprising five questions, this questionnaire assesses the number of workdays lost due to migraine over 3 months. The MIDAS focuses on migraine-induced disability affecting work/study, household duties, and leisure activities on migraine days (32). The questions measure either complete inactivity days or days with productivity reduced by 50% or more. The cumulative total of these days was subsequently categorized into four disability severity levels, as follows: Grade I (0–5 points), indicating little to no disability; Grade II (6–10 points), mild disability; Grade III (11–20 points), moderate disability; and Grade IV (21 points or more), severe disability. The MIDAS questionnaire has two components: MIDAS A, which tracks the frequency of headaches, and MIDAS B, which rates the intensity of pain on a scale of 0 (no pain) to 10 (extremely severe pain) over a three-month period.

### Pittsburgh Sleep Quality Index

The PSQI is a questionnaire that assesses total sleep time, self-reported sleep quality, and disorders present over the past month. The questionnaire contains 19 questions distributed across seven domains. Subjective sleep quality, sleep latency, sleep length, sleep activity, sleep medication usage, sleep disorders, and daytime functional performance are the seven primary elements of this scale. The sum of all component scores yields the total PSQI score, for which a decline in sleep quality is indicated by a PSQI score of >5. The Arabic version of the questionnaire was based on a previously published paper (33) and incorporated into Google Forms. Respondents were either asked to write a short text answer or select one out of four options, as per the PSQI questionnaire.

### Data analysis

Statistical Analysis was conducted using the Statistical Package for the Social Sciences (SPSS) version 29.0, released in 2022 by SPSS Inc., Chicago, IL, United States. Data are presented as the mean ± standard deviation (SD), or frequency counts and corresponding percentages. Chi-square and independent t-tests were applied to compare variables. Pearson’s correlation analysis was further used to assess the correlations between the scales. A multivariate linear regression analysis was conducted to evaluate the effect of sleep quality on migraine severity under different adjustment conditions and subgroups. Likewise, multivariate linear regression analysis was conducted to evaluate the effect of migraine severity on sleep quality. Three multivariate linear regression models were used, and all variables that were significant in the univariate regression analysis were included in the multivariate regression models. Statistical significance was set at *p* < 0.05.

## Results

### Sociodemographic data

A total of 1,567 individuals completed the questionnaire, of whom 35 were excluded for being under 18 years of age, 116 for having undergone a surgical procedure within the last 6 months, and 17 for disagreeing to participate in the study. Finally, the enrolled cohort comprised 1,399 individuals with a mean (SD) age of 27.9 ± 11.6 and BMI of 24.4 ± 6.1, most of whom were from the central region of Saudi Arabia (376 [26.9%]), were married (977 [69.8%]), and were university students or graduates (1,056 [75.5%]). A small proportion of participants were current or previous smokers (*n* = 129 [9.1%]). Moreover, almost two-thirds of the study participants had a family member complaining of headaches (898 [71.7%]).

Of the 1,399 participants included in the study, 349 had migraine according to the IHS diagnostic criteria, representing 24.95% of the total study population. Table 1 presents the sociodemographic data of the migraine patients and 1,050 controls. Notable findings included a significant sex difference, with females predominating in the migraine group [311 (89.1) vs. 758 (72.2); *p* < 0.001]. A higher proportion of individuals in the migraine group were unemployed or retired than in the control group [103 (29.5) vs. 208 (19.8); *p* < 0.001]. The average height of the migraine group was notably lower than that of the control group [160.14 (7.9) vs. 162.6 (9.2); *p* < 0.001].

**Table 1:** Sociodemographic data of the participants (*n* = 1,399).

Characteristics	Total (1399)	Migraine (349)	Control (1050)	*p*
Age (years), M (SD)	27.9 (11.6)	28.14 (13.1)	27.84 (11.1)	0.670
Gender				<0.001
Male	330 (23.6)	38 (10.9)	292 (27.8)	
Female	1,069 (76.4)	311 (89.1)	758 (72.2)	
Weight (kg)	64.5 (17.2)	63.51 (18.7)	64.92 (16.7)	0.188
Height (cm)	161.9 (8.9)	160.14 (7.9)	162.6 (9.2)	<0.001
BMI (kg/m2)	24.4 (6.1)	24.64 (6.9)	24.4 (5.8)	0.541
Region				0.161
Central region	376 (26.9)	95 (27.2)	281 (26.8)	
Eastern region	170 (12.2)	51 (14.6)	119 (11.3)	
Northern region	216 (15.4)	41 (11.7)	175 (16.7)	
Southern region	334 (23.9)	84 (24.1)	250 (23.8)	
Western region	303 (21.7)	78 (22.3)	250 (23.8)	
Educational level				0.500
High school degree or below	286 (20.4)	78 (22.3)	208 (19.8)	
University degree	1,056 (75.5)	259 (74.2)	797 (75.9)	
Master’s or PhD degree	57 (4.1)	12 (3.4)	45 (4.3)	
Marital status				0.623
Single	376 (26.9)	100 (28.7)	276 (26.3)	
Married	977 (69.8)	239 (68.5)	738 (70.3)	
Widowed	31 (2.2)	8 (2.3)	23 (2.2)	
Divorced	15 (1.1)	2 (0.6)	13 (1.2)	
Occupation				<0.001
Unemployed or retired	311 (22.2)	103 (29.5)	208 (19.8)	
Student	777 (55.5)	182 (52.1)	595 (56.7)	
Employed	311 (22.2)	64 (18.3)	247 (23.5)	
Smoking	128 (9.1)	26 (7.4)	102 (9.7)	0.204
Comorbidities*				
High blood pressure	83 (6.6)	26 (7.40)	57 (6.3)	0.468
Arterial diseases	7 (0.6)	2 (0.6)	5 (0.6)	0.967
Stomach ulcer	52 (4.2)	13 (3.7)	39 (4.3)	0.637
Asthma	77 (6.2)	24 (6.9)	53 (5.9)	0.506
Depression	115 (9.2)	26 (7.4)	89 (9.9)	0.186
Allergies of any kind	138 (11.0)	41 (11.7)	97 (10.7)	0.610
Epilepsy	10 (0.8)	1 (0.3)	9 (1.0)	0.206
Skin diseases	69 (5.5)	18 (5.2)	51 (5.6)	0.733
Taking medications for mentioned comorbidities*	216 (17.3)	52 (14.9)	164 (18.2)	0.371
Family history of headache*	898 (71.7)	250 (71.6)	648 (71.8)	0.964
Ever had a headache?	1,252 (89.5)	349 (100.0)	903 (86.0)	<0.001

*in participants who have had a headache *n* = 1,252 for migraine and *n* = 903 for control.

### MIDAS

As shown in Table 2, of the 349 participants with migraine, 83% did not miss work or school because of headaches over a period of 3 months. However, more than half of the participants did not perform household work for one or more days because of headaches. The mean total MIDAS score was 8.19 days, and was shown to be negatively affected by headaches for a duration of 3 months, while the mean MIDAS B score was 7.05. Regarding the overall MIDAS grade, more than half of the participants belonged to the little or no disability group. Moreover, only approximately 10% of migraine patients were categorized in the severe disability group.

**Table 2:** The Migraine Disability Assessment Test (MIDAS; *n* = 349).

Characteristics	Days, M(SD)
Missed work or school because of headaches (days), (*n* = 347)	0.48 (1.7)
Zero days N (%)	287 (82.7)
One or more days N (%)	60 (17.3)
Productivity at work/school reduced by half or more because of headaches (days), (*n* = 326)	2.03 (3.9)
Zero days N (%)	183 (56.1)
One or more days N (%)	143 (43.9)
Did not do household work because of headaches (days), (*n* = 324)	2.61 (4.4)
Zero days N (%)	151 (46.6)
One or more days N (%)	173 (53.4)
Productivity in household work reduced by half or more because of headaches (days), (*n* = 346)	2.21 (4.4)
Zero days N (%)	190 (54.9)
One or more days N (%)	156 (45.1)
Missed family, social or leisure activities because of headaches (days), (n = 345)	1.22 (2.1)
Zero days N (%)	205 (59.4)
One or more days N (%)	140 (40.6)
Total MIDAS score	
M (SD)	8.19 (11.11)
Median (Min-Max)	4 (0–90)
MIDAS B	
M (SD)	**7.05 (1.9)**
Median (Min-Max)	7 (1–10)
MIDAS Grade	
I, Little or No Disability	189 (54.2)
II, Mild Disability	70 (20.1)
III, Moderate Disability	56 (16.0)
IV, Severe Disability	34 (9.7)

Bold values are statistically significant.

### PSQI

As shown in Table 3, there was no significant difference in PSQI scores between the migraine and control groups. The overall cohort had a score of 6.9 (3.4), with 7.02 (3.4) for the migraine group and 6.97 (3.4) for the control group. The subjective sleep quality scores were 1.15 (0.8) in the migraine group and 1.09 (0.830) in the control group. The daily disturbance factor scores were similar between the migraine and control groups, with scores of 1.32 (0.64) and 1.36 (0.7), respectively. Overall, the PSQI score showed that most of the 813 participants had mild sleeping difficulty (56.4% migraine patients and 58.7% controls), and approximately 539 participants had moderate sleeping difficulty (40.1% migraine patients and 38% controls).

**Table 3:** Pittsburgh Sleep Quality Index (PSQI; *n* = 1,399).

Characteristics	Participants	Migraine	Control	*p*
PSQI overall subjective sleep quality score, M (SD), Median (Min-Max)	6.9 (3.4) 7 (0–19)	7.02 (3.4) 7 (0–19)	6.97 (3.4) 7 (0–18)	0.821
PSQI Components, M (SD)				
PSQI subjective sleep quality score (C1)	1.1 (0.82)	1.15 (0.8)	1.09 (0.83)	0.180
PSQI sleep latency score (C2)	1.42 (1.01)	1.47 (1.05)	1.41 (1.0)	0.360
PSQI sleep duration score (C3)	1.07 (1.05)	1.1 (1.02)	1.06 (1.06)	0.532
PSQI sleep efficiency score (C4)	0.6 (0.96)	0.59 (0.98)	0.61 (0.99)	0.838
PSQI sleep disturbances score (C5)	1.35 (0.69)	1.32 (0.64)	1.36 (0.7)	0.406
PSQI taking sleeping medications (C6)	0.4 (0.79)	0.33 (0.73)	0.42 (0.81)	0.071
PSQI daytime dysfunction score (C7)	1.13 (0.8)	1.13 (0.79)	1.12 (0.81)	0.812
PSQI Score N (%)				0.447
No sleeping difficulty	9 (0.6)	4 (1.1)	5 (0.5)	
Mild sleeping difficulty	813 (58.1)	197 (56.4)	616 (58.7)	
Moderate sleeping difficulty	539 (38.5)	140 (40.1)	399 (38.0)	
Severe sleeping difficulty	38 (2.7)	8 (2.3)	30 (2.9)	

### Association between MIDAS grade and characteristics in migraine patients

The association between MIDAS grade and various characteristics in the 349 migraine patients is presented in Table 4, of whom 259 had a little/mild MIDAS grade, and the remaining 90 migraine patients had moderate/severe disability levels. A family history of headache was significantly more common in both the little/mild (69.9%) and moderate/severe (76.7%) migraine severity groups. Another statistically significant parameter, shown in Table 4, was waking up from sleep due to headaches. More than half of those with a little/mild severity level (61.4%) did not have headache-related awakenings, whereas (35.5%) of those with the same severity grade reported sleep interruption due to headache. Conversely, approximately 50 percent of moderate/severe migraine patients (62.2%) reported waking from sleep, compared to 33.3 and 4.4% of the remaining patients who never wake up and always wake up because of headaches by (33.3%) and (4.4%), respectively. Among the medications taken for headache, ibuprofen was the only significant drug taken for headaches among both little/mild migraine patients (7.3%) and moderate/severe migraine patients (17.8%), as opposed to Panadol, Solpadeine, or other medications. Regarding whether headache improved with medications, both the little/mild (44.8%) and moderate/severe (65.6%) MIDAS groups reported that their headache sometimes improved with medications. Of the reported PSQI scores, the PSQI sleep latency score (C2; *p* = 0.02), PSQI sleep disturbance score (C5; *p* < 0.001), and PSQI daytime dysfunction score (C7; *p* = 0.022) all showed significant associations, as shown in Table 4. Finally, there was no statistically significant difference in PSQI scores across patients with different MIDAS severities.

**Table 4:** Association between MIDAS grade and variables (*n = 349*).

Characteristics [M (SD) or N (%)]	Little/Mild Disability (259)	Moderate/ Severe Disability (90)	p
Age (years)	28.42 (13.770)	27.36 (10.992)	0.509
Gender			0.753
Male	29 (11.2)	9 (10)	
Female	230 (88.8)	81 (90)	
Weight (kg)	63.89 (19.518)	62.44 (16.274)	0.528
Height (cm)	160.21 (7.998)	159.94 (7.508)	0.787
BMI (kg/m2)	24.76 (7.346)	24.31 (5.600)	0.593
Living Status			0.128
City	223 (86.1)	83 (92.2)	
Village	36 (13.9)	7 (7.8)	
Region			0.195
Central region	77 (29.7)	18 (20.0)	
Eastern region	35 (13.5)	16 (17.8)	
Northern region	32 (12.4)	9 (10.0)	
Southern region	56 (21.6)	28 (31.1)	
Western region	59 (22.8)	19 (21.1)	
Educational level			0.831
High school degree or below	58 (22.4)	20 (22.2)	
University degree	193 (74.5)	66 (73.3)	
Master’s or PhD degree	8 (3.1)	4 (4.4)	
Marital status			0.679
Single	73 (28.2)	27 (30.0)	
Married	177 (68.3)	62 (68.9)	
Widowed	7 (2.7)	1 (1.1)	
Divorced	2 (0.8)	0 (0)	
Occupation			0.553
Unemployed or retired	80 (30.9)	23 (25.6)	
Student	134 (51.7)	48 (53.3)	
Employed	45 (17.4)	19 (21.1)	
Smoking	16 (6.2)	10 (11.1)	0.125
Comorbidities*			
High blood pressure	17 (6.6)	9 (10)	0.285
Arterial diseases	2 (0.8)	0 (0)	0.403
Stomach ulcer	6 (2.3)	7 (7.8)	**0.018**
Asthma	18 (6.9)	6 (6.7)	0.927
Depression	16 (6.2)	10 (11.1)	0.125
Allergies of any kind	31 (12)	10 (11.1)	0.828
Epilepsy	1 (0.4)	0 (0)	0.555
Stroke	0 (0)	0 (0)	-
Skin diseases	13 (5)	5 (5.6)	0.843
Taking medications for mentioned comorbidities	40 (15.4)	12 (13.3)	**0.025**
Family history of headache	181 (69.9)	69 (76.7)	0.219
Severity of headache, M (Sd)	7.15 (1.919)	6.79 (1.997)	0.219
Factors trigger/worsen the headache			
Noise	131 (50.6)	53 (58.9)	0.174
Physical stress	111 (42.9)	50 (55.6)	**0.037**
Not eating regularly	87 (33.6)	43 (47.8)	**0.016**
Certain foods	13 (5)	10 (11.1)	**0.045**
Weather changes	34 (13.1)	15 (16.7)	0.405
Smells	55 (21.2)	32 (35.6)	**0.007**
Tiredness	158 (61)	61 (67.8)	0.252
Too little or too much sleep	180 (69.5)	65 (72.2)	0.626
Lights	78 (30.1)	39 (43.3)	**0.022**
Stress	106 (40.9)	46 (51.1)	0.093
Cough	10 (3.9)	10 (11.1)	**0.011**
Painkillers	6 (2.3)	4 (4.4)	0.297
Factors that relieve the headache			
Rest	148 (57.1)	58 (64.4)	0.225
Vomiting	11 (4.2)	9 (10)	**0.043**
Exercise	32 (12.4)	9 (10)	0.550
Sleeping	168 (64.9)	60 (66.7)	0.757
Sitting in a dark place	67 (25.9)	45 (50)	**<0.001**
Watching TV	2 (0.8)	2 (2.2)	0.266
Taking a warm shower	28 (10.8)	15 (16.7)	0.145
Taking a cold shower	34 (13.1)	16 (17.8)	0.278
Listening to music	3 (1.2)	4 (4.4)	0.055
Massage	52 (20.1)	22 (24.4)	0.383
Reading	3 (1.2)	1 (1.1)	0.971
Painkillers	143 (55.2)	64 (71.1)	**0.008**
Headache wakes from sleep			**<0.001**
Always	8 (3.1)	4 (4.4)	
Sometimes	92 (35.5)	56 (62.2)	
Never	159 (61.4)	30 (33.3)	
Taking any medications for headache			
Panadol	196 (75.7)	71 (78.9)	0.536
Ibuprofen	19 (7.3)	16 (17.8)	**0.004**
Triptans	0 (0)	1 (1.1)	0.089
Aspirin	2 (0.8)	2 (2.2)	0.266
Antibiotics	1 (0.4)	0 (0)	0.555
Solpadeine	0 (0)	0 (0)	–
Other medications	2 (0.8)	46 (51.1)	0.403
Headache improves with medications			**0.005**
Always	83 (32)	18 (20)	
Sometimes	116 (44.8)	59 (65.6)	
Never	6 (2.3)	2 (3.3)	
No medications used	54 (20.8)	10 (11.1)	
Practices to avoid headache			
Taking medicines	102 (39.4)	46 (51.1)	0.052
Using traditional medicines	14 (5.4)	5 (5.6)	0.957
Exercises	23(8.9)	5 (5.6)	0.317
Rest	57 (22.0)	26 (28.9)	0.187
PSQI, M (SD), Median (Min-Max)	6.81 (3.414) 6.00 (0–19)	7.61 (3.179) 7.00 (0–17)	0.053
PSQI subjective sleep quality score (C1)	1.13 (0.828)	1.23 (0.704)	0.279
PSQI sleep latency score (C2)	1.39 (1.067)	1.69 (0.979)	**0.020**
PSQI sleep duration score (C3)	1.15 (1.030)	0.97 (0.988)	0.154
PSQI sleep efficiency score (C4)	0.61 (0.988)	0.55 (0.945)	0.658
PSQI sleep disturbances score (C5)	1.25 (0.598)	1.53 (0.722)	**<0.001**
PSQI taking sleeping medications (C6)	0.31 (0.713)	0.39 (0.789)	0.373
PSQI daytime dysfunction score (C7)	1.08 (0.818)	1.30 (0.710)	**0.022**
PSQI Score			0.533
No sleeping difficulty	3 (1.2)	1 (1.1)	
Mild sleeping difficulty	152 (58.7)	45 (50.0)	
Moderate sleeping difficulty	98 (37.8)	42 (46.7)	
Severe sleeping difficulty	6 (2.3)	2 (2.2)	

Bold values are statistically significant.

### Association between PSQI and characteristics in migraine patients

Table 5 presents the association between the PSQI and migraine characteristics in 349 patients with migraine. Accordingly, 201 patients with migraine had no/mild PSQI grades and 148 had moderate/severe levels of sleep difficulty. Statistical analysis of the sociodemographic variables revealed no statistical differences in PSQI grade. High blood pressure was the only statistically significant comorbidity among patients with migraine. Statistically significant differences were further observed between those with no or mild sleeping difficulties, and those with moderate or severe sleeping difficulties taking medications for the comorbidities listed in Table 5. Among migraine patients in both PSQI groups, Table 5 shows the factors that trigger/worsen headaches. Overall, a lack of or excessive sleep was the most common migraine trigger for people with no/mild sleeping difficulties (61.7%) and moderate/severe sleeping difficulties (81.8%). This was followed by fatigue, stress, and certain smells with comparative frequencies in both the no/mild and moderate/severe PSQI groups, as shown in Table 5. Both PSQI groups reported significant results, indicating that both suffered from sleep disturbances as a result of headaches, albeit with differences in frequency. The no/mild sleeping difficulty group reported almost never waking up from sleep because of headaches. However, the moderate/severe group occasionally awoke from sleep. On the PSQI, 33.8% of those with no or mild sleeping difficulties reported occasional disturbances during sleep, compared with 54.1% of those with moderate or severe sleeping difficulties. In the no/mild sleep difficulty group, 3% reported always experiencing disturbed sleep due to headaches, whereas the moderate/severe sleep difficulty group reported a PSQI score of 4.1%. Medications improving headaches have been shown to be statistically significant in both PSQI groups, with the moderate to severe group reporting that using medications sometimes improved the headache (60.8%) With respect to the means to avoid headaches, taking medications demonstrated a statistical significance in both the no/mild sleeping difficulty (36.8%) and the moderate/ severe sleeping difficulty (50%) groups, making it the only statistically significant prophylactic strategy employed compared to the other practices. In this study, PSQI severity exhibited a significant *p*-value of <0.001. MIDAS scores and grades had *p*-values of 0.002 and 0.003, respectively, indicating that MIDAS grades and sleep quality were related.

**Table 5:** Association between sleep quality and variables in migraine patients (*n* = 349).

Characteristics [M (SD) or N (%)]	No/mild sleeping difficulty (201)	Moderate /severe sleeping difficulty (148)	*p*
Age (years)	28.33 (14.399)	27.89 (11.147)	0.759
Gender			0.316
Male	19 (9.5)	19 (12.8)	
Female	182 (90.5)	129 (87.2)	
Weight (kg)	62.30 (16.348)	65.15 (21.467)	0.160
Height (cm)	159.78 (8.131)	160.62 (7.490)	0.324
BMI (kg/m2)	24.29 (6.278)	25.11 (7.720)	0.276
Living Status			0.801
City	177 (88.1)	129 (87.2)	
Village	24 (11.9)	19 (12.8)	
Region			0.773
Central region	52 (25.9)	43 (29.1)	
Eastern region	32 (15.9)	19 (12.8)	
Northern region	21 (10.4)	20 (13.5)	
Southern region	49 (24.4)	35 (23.6)	
Western region	47 (23.4)	31 (20.9)	
Educational level			0.109
High school degree or below	37 (18.4)	41 (27.7)	
University degree	156 (77.6)	103 (69.6)	
Master’s or PhD degree	8 (4.0)	4 (2.7)	
Marital status			0.693
Single	59 (29.4)	41 (27.7)	
Married	138 (68.7)	101 (68.2)	
Widowed	3 (1.5)	5 (3.4)	
Divorced	1 (0.5)	1 (0.7)	
Occupation			0.178
Unemployed or retired	52 (25.9)	51 (34.5)	
Student	108 (53.7)	74 (50.0)	
Employed	41 (20.4)	23 (15.5)	
Smoking	11 (5.5)	15 (10.1)	0.101
Comorbidities			
High blood pressure	9 (4.5)	17 (11.5)	**0.014**
Arterial diseases	0 (0)	2 (1.4)	0.098
Stomach ulcer	7 (3.5)	6 (4.1)	0.781
Asthma	11 (5.5)	13 (8.8)	0.227
Depression	12 (6)	14 (9.5)	0.220
Allergies of any kind	19 (9.5)	22 (14.9)	0.121
Epilepsy	0 (0)	1 (0.7)	0.243
Stroke	0 (0)	0 (0)	–
Skin diseases	7 (3.5)	11 (7.4)	0.099
Taking medications for mentioned comorbidities	27 (13.4)	25 (16.9)	**0.012**
Family history of headache	142 (70.6)	108 (73)	0.634
Severity of headache, M (Sd)	7.07 (1.963)	7.03 (1.922)	0.865
Factors trigger/worsen the headache			
Noise	100 (49.8)	84 (56.8)	0.195
Physical stress	87 (43.3)	74 (50)	0.214
Not eating regularly	67 (33.3)	63 (42.6)	0.078
Certain foods	13 (6.5)	10 (6.8)	0.914
Weather changes	22 (10.9)	27 (18.2)	0.052
Smells	42 (20.9)	45 (30.4)	**0.042**
Tiredness	110 (54.7)	109 (73.6)	**<0.001**
Too little or too much sleep	124 (61.7)	121 (81.8)	**<0.001**
Lights	61 (30.3)	56 (37.8)	0.143
Stress	71 (35.3)	81 (54.7)	**<0.001**
Cough	11 (5.5)	9 (6.1)	0.809
Painkillers	5 (2.5)	5 (3.4)	0.622
Factors that relieve the headache			
Rest	118 (58.7)	88 (59.5)	0.888
Vomiting	12 (6)	8 (5.4)	0.823
Excercise	23 (11.4)	18 (12.2)	0.837
Sleeping	131 (65.2)	97 (65.5)	0.943
Sitting in a dark place	60 (29.9)	52 (35.1)	0.296
Watching TV	3 (1.5)	1 (0.7)	0.479
Taking a warm shower	21 (10.4)	22 (14.9)	0.805
Taking a cold shower	28 (13.9)	22 (14.9)	0.215
Listening to music	4 (2)	3 (2)	0.981
Massage	38 (18.9)	36 (24.3)	0.221
Reading	1 (0.5)	3 (2)	0.185
Painkillers	115 (57.2)	92 (62.2)	0.352
Headache wakes from sleep			**<0.001**
Always	6 (3.0)	6 (4.1)	
Sometimes	68 (33.8)	80 (54.1)	
Never	127 (63.2)	62 (41.9)	
Taking any medications for headache			
Panadol	152 (75.6)	115 (77.7)	0.650
Ibuprofen	17 (8.5)	18 (12.2)	0.255
Triptans	0(0)	1 (0.7)	0.243
Aspirin	2 (1)	2 (1.4)	0.757
Antibiotics	1 (0.5)	0 (0)	0.390
Solpadeine	0 (0)	0 (0)	–
Other medications	0 (0)	2 (1.4)	0.098
Headache improves with medications			**0.002**
Always	72 (35.8)	29 (19.6)	
Sometimes	85 (42.3)	90 (60.8)	
Never	4 (2.0)	5 (3.4)	
No medications used	40 (19.9)	24 (16.2)	
Practices to avoid headache			
Taking medicines	74 (36.8)	74 (50)	**0.014**
Using traditional medicines	9 (4.5)	10 (6.8)	0.354
Exercises	16 (8)	12 (8.1)	0.960
Rest	44 (21.9)	39 (26.4)	0.333
Total MIDAS score, M (SD), Median (Min-Max)	6.58 (9.034) 3.00 (0–60)	10.37 (13.154) 7 (0–90)	**0.002**
MIDAS grade			**0.003**
I, Little or No Disability	124 (61.7)	65 (43.9)	
II, Mild Disability	31 (15.4)	39 (26.4)	
III, Moderate Disability	32 (15.9)	24 (16.2)	
IV, Severe Disability	14 (7.0)	20 (13.5)	
MIDAS B, M (SD)	7.07 (1.963)	7.03 (1.922)	0.865

Bold values are statistically significant.

### Correlation of PSQI components with MIDAS

**Figure 1:**
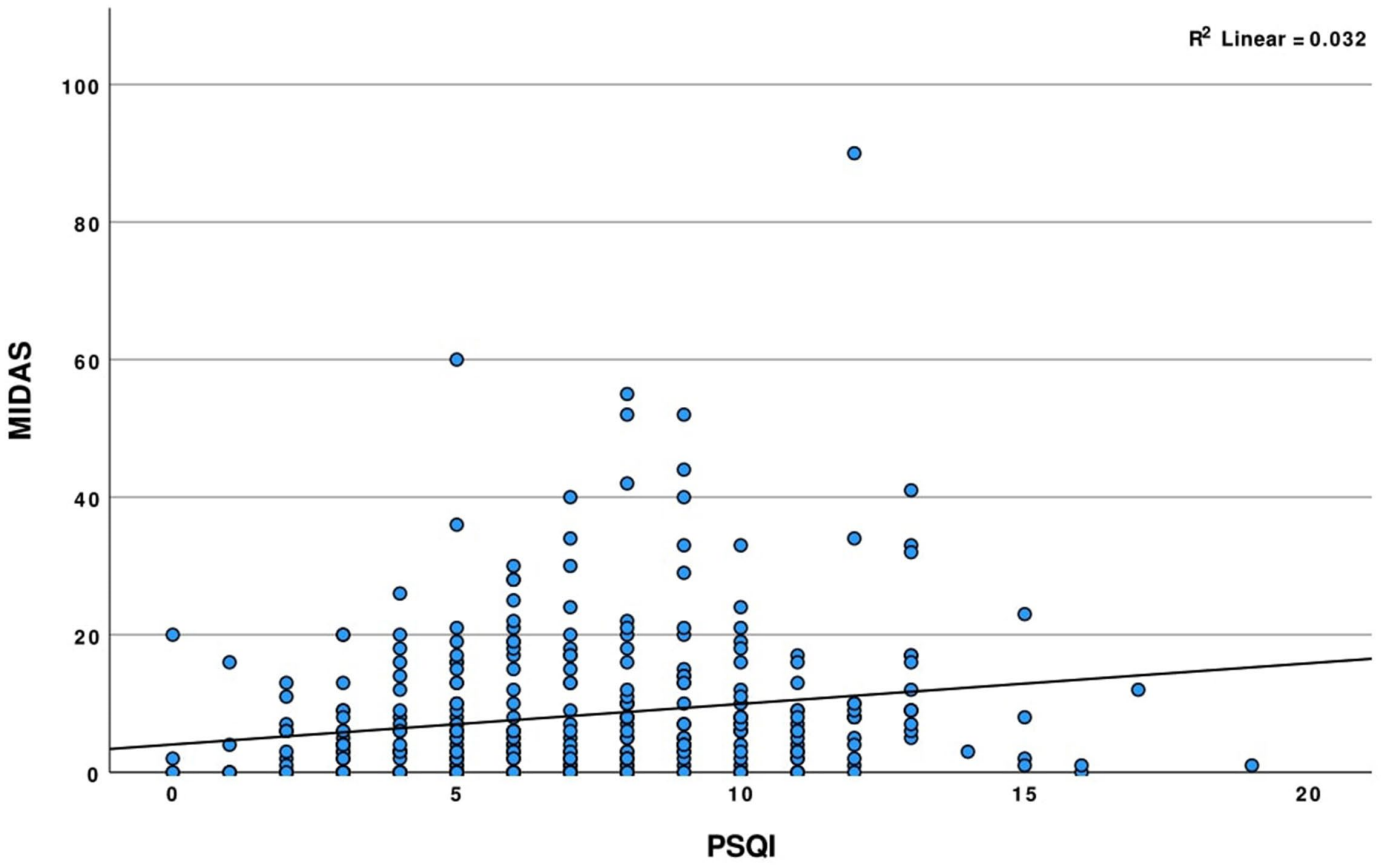
Scatter plot illustrating the correlation between MIDAS and PSQI scores among participants with migraine.

A positive correlation was found between the PSQI and MIDAS scores in migraine patients, which was statistically significant (*r* = 0.18, *p* < 0.001) as shown in Table 6. The MIDAS was significantly correlated positively with the following PSQI components: subjective sleep quality (*r* = 0.11, *p* = 0.047), sleep latency (*r* = 0.19, *p* < 0.001), sleep disturbance (*r* = 0.23, *p* < 0.001), and daytime dysfunction (*r* = 0.2, *p* < 0.001). The Pearson correlation test did not reveal any association between the PSQI and its components with MIDAS B (Figure 1).

**Table 6:** Correlation of PSQI total and 7 component scores with MIDAS.

Variable	MIDAS				MIDAS B			
	Correlation	Lower CI	Upper CI	*p*	Correlation	Lower CI	Upper CI	*p*
C1	0.107	0.002	0.209	**0.047**	−0.013	−0.118	0.092	0.811
C2	0.194	0.090	0.293	**<0.001**	0.093	−0.012	0.196	0.083
C3	−0.028	−0.134	0.078	0.602	0.013	−0.093	0.119	0.811
C4	−0.026	−0.136	0.084	0.642	−0.069	−0.178	0.042	0.222
C5	0.225	0.122	0.322	**<0.001**	−0.039	−0.144	0.066	0.464
C6	0.059	−0.046	0.163	0.269	0.012	−0.094	0.116	0.829
C7	0.199	0.096	0.298	**<0.001**	−0.005	−0.110	0.100	0.932
PSQI score	0.179	0.075	0.279	**<0.001**	0.005	−0.100	0.110	0.931

Bold values are statistically significant.

### Multivariate linear regression analysis of sleep quality on migraine severity

Multivariate linear regression analysis of sleep quality on migraine severity, represented as the MIDAS, is shown in Table 7. In total, three models were constructed based on the results of the univariate regression analysis, literature, and the clinical significance of these factors. This analysis revealed that the PSQI score under no-adjustment conditions, as in Model 1, was insignificant in predicting migraine severity. After adjusting for smoking, as in Model 2, a significant association between smoking and migraine severity was detected, whereby an incremental increase in smoking worsened migraine severity by 0.108 (beta coefficient = −0.108, *p* < 0.042). Model 3 further illustrated the significance of smoking as a predictor of migraine severity, despite adjusting for age, sex, and BMI.

**Table 7:** Multivariate linear regression analysis of sleep quality on the severity of migraine (MIDAS).

Model	Variable	Unstandardized β	SE	Standardized β	t	*p*-value	(95%CI)
Model 1	PSQI score	0.408	0.305	0.124	1.338	0.182	(−0.192, 1.008)
	Poor sleep quality	1.510	2.076	0.067	0.727	0.468	(−2.574, 5.593)
Model 2	PSQI score	0.397	0.304	0.120	1.306	0.192	(−0.201, 0.994)
	Poor sleep quality	1.362	2.068	0.061	0.659	0.511	(−2.705, 5.430)
	Smoking	−4.553	2.233	−0.108	−2.039	**0.042**	(−8.944, −0.162)
Model 3	PSQI score	0.391	0.306	0.119	1.279	0.202	(−0.210, 0.993)
	Poor sleep quality	1.470	2.089	0.065	0.704	0.482	(−2.640, 5.579)
	Smoking	−5.016	2.311	−0.119	−2.171	**0.031**	(−9.562, −0.471)
	Age	−0.028	0.047	−0.032	−0.582	0.561	(−0.121, 0.066)
	Gender	1.654	1.963	0.046	0.842	0.400	(−2.207, 5.515)
	BMI	−0.097	0.090	−0.061	−1.078	0.282	(−0.275, 0.080)

Model 1 unadjusted. Model 2 adjusted for smoking. Model 3 adjusted for age, gender, BMI, and smoking. Bold values are statistically significant.

### Multivariate linear regression analysis of migraine severity on sleep quality

Table 8 shows the results of the multivariate linear regression analysis. The MIDAS score was the only significant factor retained after univariate regression analysis for all demographics. Hence, all variables were adjusted in Model 2. A significant association between migraine severity and poor sleep quality in patients with migraine was detected in Models 1–2. For every 1-point increase in the MIDAS score (i.e., 1 day missed due to migraine headache), the PSQI score (i.e., sleeping difficulty) increased by 0.179 (beta coefficient = 0.179, *p* < 0.001) in all migraine patients.

**Table 8:** Multivariate linear regression analysis of severity of migraine on the sleep quality (PSQI).

Model	Variable	Unstandardized β	SE	Standardized β	t	*p*-value	(95%CI)
Model 1	MIDAS score	0.054	0.016	0.179	3.387	**<0.001**	(0.023, 0.086)
	Moderate/Severe Migraine	0.796	0.411	0.104	1.940	0.053	(−0.011,1.60)
Model 2	MIDAS score	0.073	0.025	0.240	2.859	**0.005**	(0.023, 0.123)
	Moderate/Severe Migraine	−0.654	0.643	−0.085	−1.017	0.310	(−1.918, 0.610)

Model 1 unadjusted. Model 2 adjusted for age, gender, BMI, and smoking. Bold values are statistically significant.

## Discussion

The relationship between migraine and sleep disturbances is complex (34). In this comparative study analyzing the association between migraine and sleep disturbance, a significant positive correlation was found between migraine severity and poor sleep quality among migraine patients. Moreover, our findings indicated that migraine severity directly influences sleep quality, emphasizing the need for integrated treatment approaches that address both sleep and migraine management. Such insights could aid in the development of more effective therapeutic strategies for improving the quality of life of individuals with migraine.

Stress is one of the most common triggers of migraine attacks, with or without aura. Although the influence of stress on the burden of migraine remains unclear, our findings demonstrate that it is more common in migraine patients who score higher on the MIDAS, indicating a possible causal relationship (35). Several studies have previously extensively investigated the influence of diet and eating habits on migraine; however, a definitive association has yet to be confirmed. Nevertheless, our findings indicate that certain foods, as well as irregular eating, can significantly influence the impact of migraine on quality of life. Additionally, food avoidance and other unhealthy eating habits are associated with stress, which negatively influences MIDAS scores (36). Pain is associated with the disruption of normal functioning in individuals with various comorbidities (37). According to Kim et al., distorted pain perception may be related to migraine-related disability. Consequently, our study showed that patients with migraine with better MIDAS scores demonstrated an ability to relieve pain (38). Headaches that trigger awakening from sleep showed a significant negative influence on MIDAS scores, which could be explained by the association between decreased REM sleep duration and the worsening of cutaneous allodynia symptoms that occur during a migraine attack, which could further reduce an individual’s quality of life (29). Finally, our study found that those with severe migraine disability had trouble falling asleep, sleep disturbances, and daytime dysfunction, further supporting the overall conclusion of this study.

The bidirectional nature of migraine and sleep quality, and the relationship thereof, have both been considerably scrutinized by researchers to uncover how sleep quality and migraine intertwine in a two-faceted manner (39–41). One case–control study in China assessed the association between migraine and sleep quality, noting that, despite the lack of a significant linear trend between poor sleep quality and MIDAS score, migraine patients with poor sleep quality showed higher MIDAS scores than those with good sleep quality (39). As such, their study successfully highlighted the unidirectional effect of sleep quality on migraine severity, which is consistent with the findings of our study. Further supporting the association between PSQI and MIDAS scores, another study showed that the MIDAS score and migraine frequency both exhibit a positive correlation with poor sleep quality (40). In contrast to the aforementioned unidirectional relationship, the extent of disability in patients with chronic migraine is thought to predict poor sleep quality (41). Contrary to expectations, our study failed to establish any bidirectional correlation between the MIDAS and PSQI scores, and a significant difference between the two MIDAS groups in terms of PSQI score was not observed, regardless of the MIDAS grade group.

The theory that smoking could be implicated in the precipitation and worsening of migraine has been investigated in several studies. Interestingly, the findings of these studies were inconsistent, as some studies found an association between smoking and migraine (42–45), whereas others found no association (46). For example, an association was identified between smoking habits and the prevalence of migraine. Smokers who consumed more cigarettes per day were found to have higher odds of experiencing migraine headaches, indicating that increased smoking frequency heightened the risk (42). Migraine patients with a high frequency of attacks (i.e., more than one attack per month) were significantly more prevalent in smokers (77%) than in nonsmokers (56%). Additionally, among students who were both smokers and migraine patients, 71% thought that smoking worsened migraine, while 59% thought that smoking precipitated migraine attacks, which agrees with our research findings (43). Furthermore, smoking >10 cigarettes per day was significantly related to migraine, with a higher severity of headaches reported among smokers than among nonsmokers (45).

Despite a vast wealth of scientific evidence, the correlation between sleep quality and migraine severity remains equivocal (34). One meta-analysis of 21 studies examining the relationship between the PSQI and MIDAS identified only a small insignificant effect size, rendering such a relationship non-existent (29). In the multivariate regression analysis of a study conducted on 120 migraine patients in Turkey, PSQI was found to be a significant determinant of MIDAS, in which a higher PSQI score resulted in more severe migraine attacks (47). Similarly, Safak et al. conducted a study on 150 migraine patients, in which they found that the PSQI was higher among migraine participants than among their non-migraine counterparts (48). With that said, the present study, having the largest sample size of migraine patients among the above-mentioned studies, illustrated that the PSQI score, in the presence or absence of adjustment conditions, cannot be confidently recommended as a tool to predict migraine severity.

A scientific study conducted in China with a sample of 134 individuals with migraine revealed a strong influence of sleep on the migraine-related burden between sleep quality and migraine-related burden (39). Previous studies have suggested that sleep, as the culprit, plays a significant role in increasing MIDAS scores, and may further contribute to the occurrence of migraine attacks, while other studies have suggested a bidirectional relationship between sleep quality and migraine (16, 49). Similarly, one systematic review published in 2020 acknowledged that despite extensive research, the exact nature of this connection remains elusive, with migraine potentially arising from sleep disturbances and sleep disruptions potentially triggering migraine (50). However, our study provides further evidence of the significant impact of migraine severity, as measured by the MIDAS score, on sleep quality. This finding supports the notion that migraine acts as an obstacle to achieving a restful sleep pattern, and confirms the directionality of this relationship.

All studies have limitations, and our study is no exception. One limitation of this study was that all components of sleep quality are merely subjective self-assessments without utilizing tools, such as polysomnography (PSG), to assess the parameters of sleep. Similarly, MIDAS was advantageous in addressing paid work or school/work, leisure time, and household responsibilities, which are all aspects that can be affected by migraine. A disadvantage of MIDAS, however, was for patients who do not vividly recall the necessary components that are assessed by MIDAS, such as the severity or the frequency of certain questions, thus leaving room for under or overestimation. Moreover, respiratory and sleep disorders, like COVID-19 and Obstructive Sleep Apnea (OSA), which can be potential influencers of sleep and headache, were not investigated (51, 52). A further limitation is that we collected the data of our participants through surveys; therefore, our survey was not accessible to individuals without internet access or devices necessary for participation. As with many cross-sectional studies, it is imperative to be aware of the limitations of the results in predicting outcomes. For example, establishing a true cause-and-effect relationship without longitudinal data is impossible. Nevertheless, this study provided considerable insight into the direct relationship between migraine severity and sleep quality.

## Conclusion

The findings of this study suggest a notable correlation between poor sleep quality and increased migraine severity. Migraine severity is a predictor of poor sleep quality. This study underscores the need for integrated treatment approaches that combine migraine management and strategies to improve sleep quality. Future research should investigate the causal mechanisms and explore whether interventions targeting sleep disturbance can effectively reduce migraine severity. Smoking negatively affected migraine severity. As such, a definite understanding of the impact of lifestyle factors on migraine severity is crucial for enhancing the quality of life of migraine patients.

## Data Availability

The original contributions presented in the study are included in the article/supplementary material, further inquiries can be directed to the corresponding author.

## Footnotes

^1^ https://epitools.ausvet.com.au/
